# 2024 joint* BMC Ecology and Evolution* and BMC Zoology image competition: the winning images

**DOI:** 10.1186/s12862-024-02291-6

**Published:** 2024-08-16

**Authors:** Jennifer Harman, Marie-Therese Nödl, Brock Fenton, Christy A. Hipsley, David A. Liberles, Edward Narayan, Josef Settele, Arne Traulsen

**Affiliations:** 1 BMC Series, Springer Nature, London, UK; 2 University of Western Ontario, London, ON Canada; 3 University of Copenhagen, Copenhagen, Denmark; 4 Temple University, Pennsylvania, USA; 5 The University of Queensland, Brisbane, Australia; 6 Helmholtz -Centre for Environmental Research – UFZ, Leipzig, Germany; 7 Max-Planck Institute for Evolutionary Biology, Schleswig-Holstein, Germany

## Abstract

In 2024, researchers from around the world entered the joint *BMC Ecology and Evolution* and *BMC Zoology* image competition. The photos, a celebration of the wonders and mysteries of the natural world, emphasise the importance of protecting life on our planet. This editorial announces the winning images chosen by the Editors and senior members of the journal’s editorial boards.

## Introduction

We are thrilled to announce the winning images of our joint *BMC Ecology and Evolution and BMC Zoology* photography competition. Like in previous years [1–10], we received a spectacular collection of images from ecologists, evolutionary biologists, zoologists and palaeontologists from around the globe, including Australia, Greece, India, Portugal, South Africa, Sweden and the USA. *BMC Ecology and Evolution* and *BMC Zoology* invited anyone affiliated with a research institution to submit to one of the following four categories: ‘Research in Action’, ‘Relationships in nature’, ‘Protecting our planet’, and ‘Life close-up’.

Our Senior Editorial Board Members lent their expertise to judge the submissions, selecting the overall winner, best image, and runner-up from each category. The board members assessed the scientific stories behind the photos and their artistic merit.

### Overall winner

Marine biologist Jorge Fontes captured the winning photograph. Jorge works at Okeanos-UAc, Institute of Marine Sciences, and is involved in a project to study the impact of fishing on the largest animals in the oceans. "The world's largest fish, the whale shark, is a gentle giant found in warm tropical and subtropical waters globally," explains Fontes. "It likes to eat plankton, which it filters through its wide mouth. During warm summers, many adult whale sharks gather near the Azores. However, because these waters are not very productive in the summer, there isn't much plankton for them to eat. Instead, the slow-moving whale sharks feed on snipefish, herded into tight groups at the surface by large schools of speedy bluefin and smaller tropical tunas, leading to a feeding frenzy. With the baitfish gathered together, the whale sharks use powerful suction to fill their huge mouths with food. This feeding partnership between sharks and tunas is rare elsewhere but common in these islands when both are present. To better understand this unique behaviour and the impact of local tuna fisheries, we have tagged whale sharks with advanced trackers (equipped with accelerometers, cameras, and sensors to measure location, pressure, and temperature)".

**Fig. 1:**
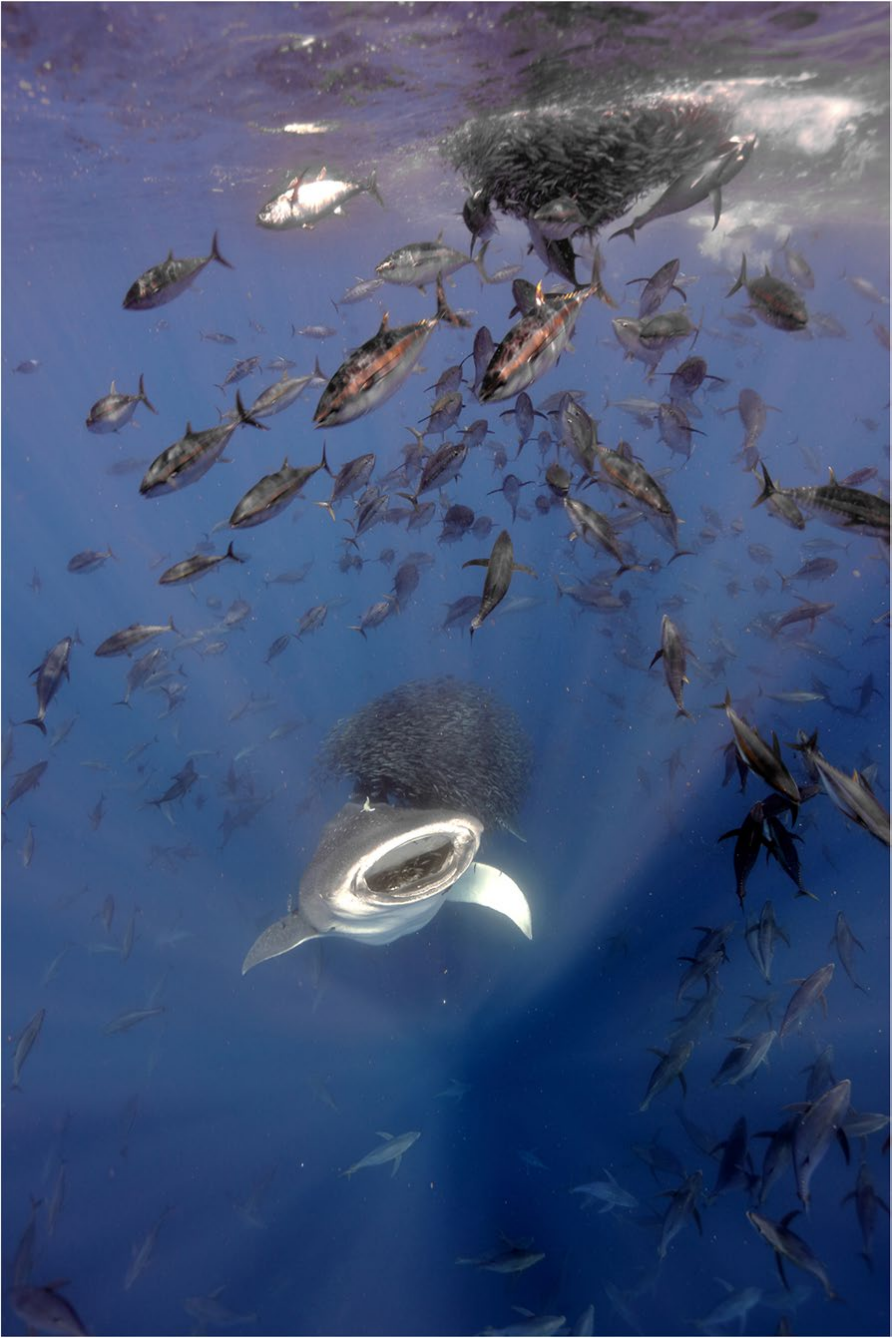
Feeding frenzy: The world's largest fish, a slow-moving whale shark, feeds on snipefish herded into bait balls by agile tuna in the Azores. Attribution: Jorge Fontes

Marie Therese Noedl, the Editor of *BMC Zoology*, says, "This beautiful picture speaks to a diverse audience and shows the interconnectedness between different species in marine food webs". Jennifer Harman, the Editor of *BMC Ecology and Evolution,* adds, “It tells a fascinating story and highlights the need for further research to understand the impact of human activities, such as the tuna industry, on whale sharks.” *BMC Ecology and Evolution* senior Editorial Board Member Christy Hipsley, comments, “You can almost hear the chaos of this scene, juxtaposed with the slow glide of the whale shark. Swirls of fish navigate to avoid each others’ mouths, dangerously distracting them from the largest one of all.” (Fig. 1).

### Research in action: best in category

**Fig. 2:**
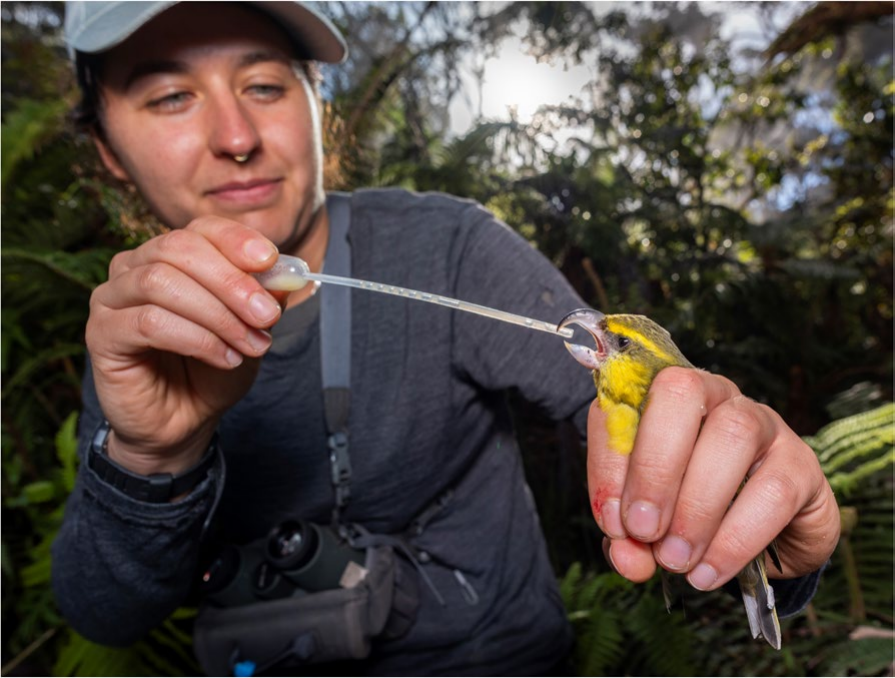
A Few Drops of Medicine: An ornithologist feeds a critically endangered Kiwikiu electrolytes and protein to prepare it for its journey to the Maui Bird Conservation Center. Attribution: Ryan Wagner

Ryan Wagner, a conservation biologist in the School of Biological Sciences at Washington State University, won best in category for "Research in action". The photograph features an ornithologist carefully feeding a few droplets of medicine to a Kiwikiu. "A single mosquito bite can kill a Kiwikiu, one of the most endangered birds in the world, with only 130 remaining. Following the introduction of mosquitoes in the 1800s, which carry deadly avian malaria, native Maui birds have found refuge on the high, cold slopes of the Haleakala volcano. However, as climate change warms the islands, mosquitoes have reached previously inaccessible elevations, killing birds. In 2024, the Maui Forest Birds Recovery Project (MFBRP) made the difficult decision to catch several pairs of Kiwikiu to create a captive population. As shown in the photo, medicine administered by ornithologists provides a boost of electrolytes and protein to strengthen the bird for a quick helicopter flight to the Maui Bird Conservation Center. There, it will be treated for malaria and join a captive breeding program to help save the species from extinction", explains Ryan. *BMC Zoology* Senior Editorial Board Member Edward Narayan comments, "The photo captures the huge challenge that humanity faces to save biodiversity, and this can only be made possible with the helping hand of scientific researchers that work towards reversing the impacts of climate change on rare and endemic wildlife species such as the Kiwikiu.” (Fig. 2).

### Research in action: runner-up

**Fig. 3:**
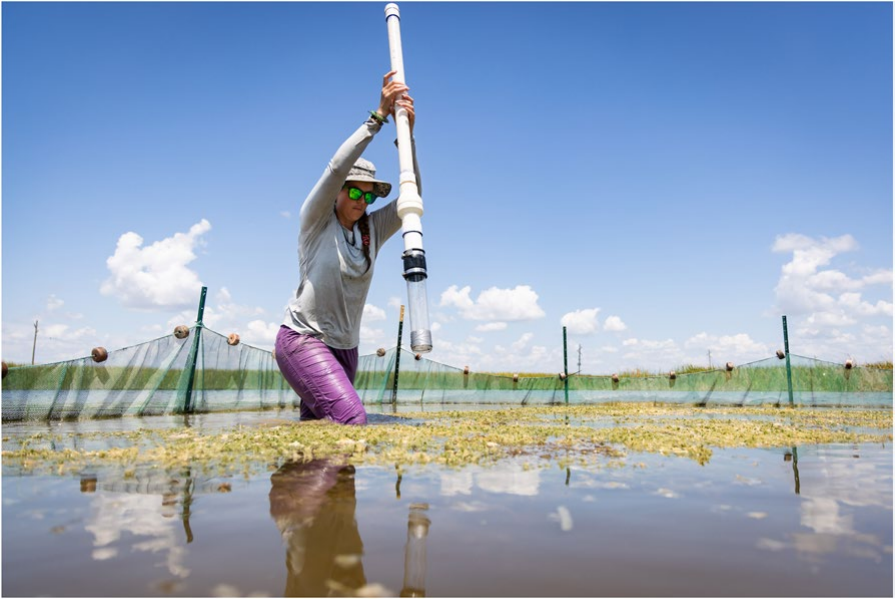
Restoration of the Everglades: A postdoctoral researcher investigates the role fauna play in phosphorus dynamics. Attribution: Brandon Güell

The runner-up of the ‘Research in action’ category was taken by Brandon Güell, a Postdoctoral Research Associate working in the Institute of Environment at Florida International University. The presence of phosphorus in agricultural and stormwater runoff has significantly degraded the water quality in the Florida Everglades, posing a threat to the extraordinary wildlife that resides there. High phosphorus levels can stimulate the growth of specific plant species, decreasing the diversity of native plants and supporting the growth of some invasive species that outcompete native Everglade plants and threaten the wildlife they support. High phosphorus levels can also trigger massive algae blooms that deplete dissolved oxygen in the water, choking aquatic life. The state and federal governments have recognized the importance of this delicate subtropical wetland and responded by funding the largest and most expensive ecosystem restoration project in the world—the restoration of the Everglades. Güell explains here, "Postdoctoral Research Associate Dr. Janelle Goeke collects cores to look at aquatic plant biomass in experimental fish exclosures in the Everglades Stormwater Treatment Areas on the border of the Everglades Protection Area. These wetlands were constructed to remove excess phosphorus from water flowing to the Everglades and Goeke's research at Florida International University aims to understand the role fauna play in the phosphorus dynamics of the system and if management of fish populations might be an effective restoration tool. Her exclosure experiment assessed whether removing fish from an area increases vegetation growth, which supports long-term storage of phosphorus.” (Fig. 3).

### Relationships in nature: best in category

**Fig. 4:**
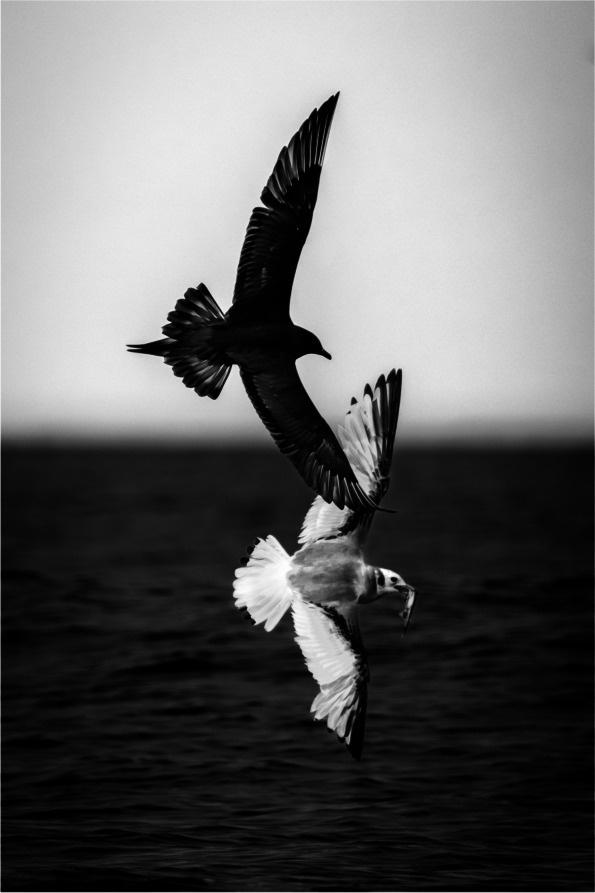
Chasing the fish: An Arctic Skua mimics the flight of a Black-legged Kittiwake to steal its fish. Attribution: Alwin Hardenbol

The winner of the 'Relationships in nature' category captured an Arctic Skua (*Stercorarius parasiticus*) mimicking the flight pattern of a Black-legged Kittiwake (*Rissa tridactyla*) in an attempt to steal the Kittiwake's fish. The photo was submitted by Alwin Hardenbol, a postdoctoral researcher in the Department of Ecology at the Swedish University of Agricultural Sciences. Alwin comments, "This behaviour, where one animal deliberately takes food from another, was observed from a boat near Vardö, Norway. Arctic Skuas often exhibit this type of behaviour, known as kleptoparasitism, in large numbers. While in Norway, I observed Humpback whales bringing fish to the surface, which the Kittiwakes and other gulls caught. The Skuas then attempted to steal the fish from them. All of the Skuas' attempts I observed were successful, demonstrating their efficiency in this relationship." *BMC Ecology and Evolution* senior Editorial Board Member David Liberles comments, "This is a really compelling image showing a clear ecological relationship—competition." (Fig. 4).

### Relationships in nature: runner-up

**Fig. 5:**
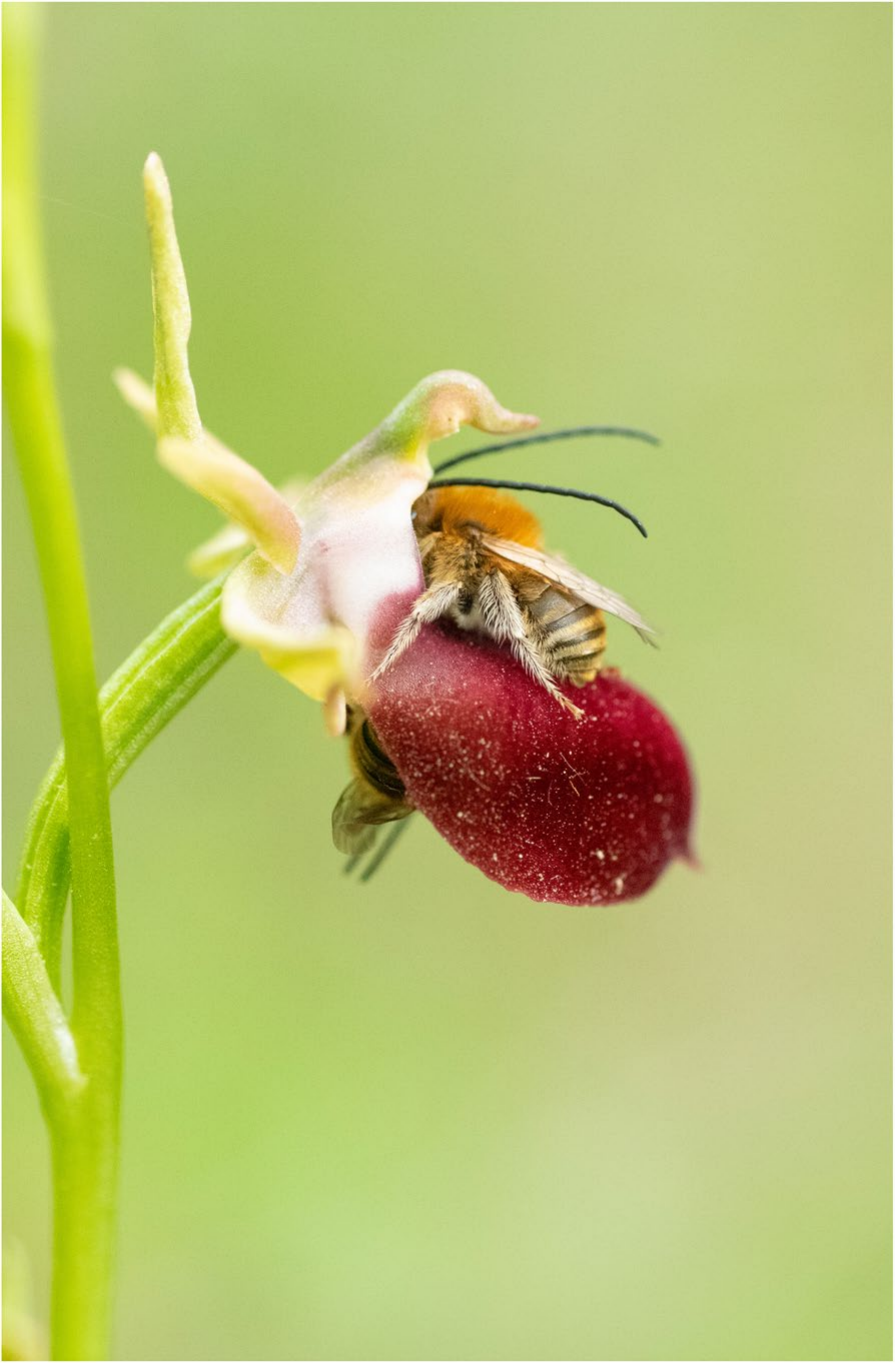
Sleeping on red velvet: A plant-pollinator interaction between a Helen's Bee orchid and two Eucerini bees. Attribution: Martha Charitonidou

Martha Charitonidou, a Postdoctoral Researcher from the Laboratory of Ecology at the University of Ioannina, submitted the runner-up for 'Relationships in Nature'. The photo captures a plant-pollinator interaction between a Helen's Bee orchid (*Ophrys helenae*), found primarily in Greece and other Balkan countries, and a long-horned bee (genus *Eucera*). "Unlike the majority of Ophrys orchids, which produce flowers imitating female insects, Helen's Bee orchid is the only species in this genus known to use shelter mimicry as its pollination strategy," Charitonidou explains. "Helen's Bee orchid is one of the most easily recognizable members of the genus Ophrys due to its large flowers with a specialised, burgundy-coloured petal (labellum), which in the bee visible spectrum looks like a black hole on the ground that a bee would use to seek shelter. As shown in this photo, male Eucerini bees can be seen crawling into Helen Bee orchid flowers in search of shelter during early morning or late evening hours." (Fig. 5).

### Protecting our planet: best in category

**Fig. 6:**
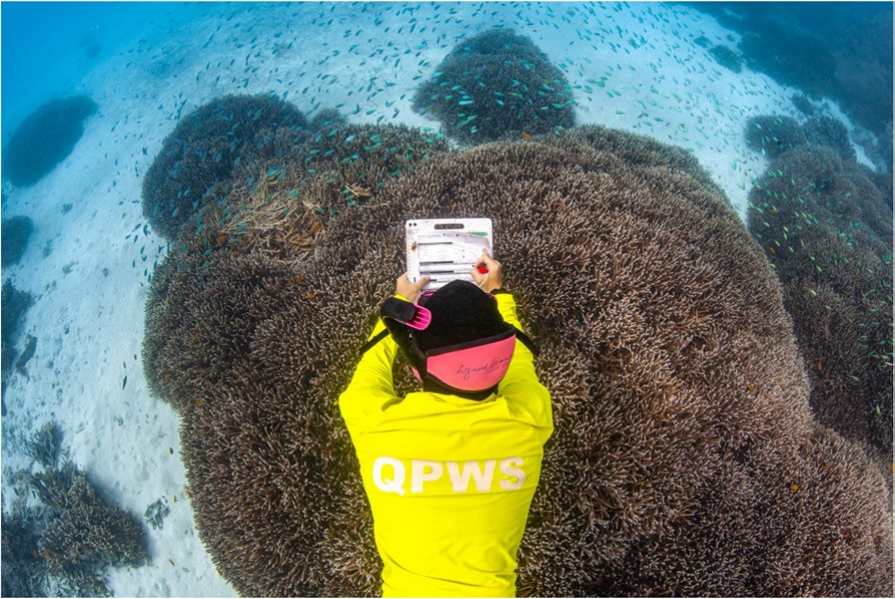
Coral health monitoring at the Great Barrier Reef: A park ranger assesses coral health at Lady Musgrave Reef in the southern Great Barrier Reef.  Attribution: Victor Huertas

Victor Huertas, a Postdoctoral Research Fellow at the College of Science and Engineering at James Cook University in Australia, captured the winning image for the ‘Protecting our planet’ category. The photo beautifully captures a park ranger assessing coral health at Lady Musgrave Reef in the southern Great Barrier Reef. The Southern Great Barrier Reef, including Lady Musgrave Reef, escaped previous mass coral bleaching events brought on by anthropogenic climate change. Corals bleach when stressed, causing them to expel the colourful photosynthetic algae that live inside them and supply them with nutrients. The Australian government's Great Barrier Reef Marine Park Authority recently reported that this summer the UNESCO World Heritage-listed reef system has experienced widespread coral bleaching, the fifth mass bleaching episode in the Great Barrier Reef since 2016*.* Victor explains, "Equipped with a slate and data sheets, the ranger examines coral colonies, documenting their condition. Monitoring coral health is important for understanding and preserving this delicate ecosystem. The vibrant reef and the marine life it harbours emphasise the beauty and ecological significance of the Great Barrier Reef and the need for ongoing conservation efforts (Fig. 6).

### Protecting our planet: runner-up

**Fig. 7:**
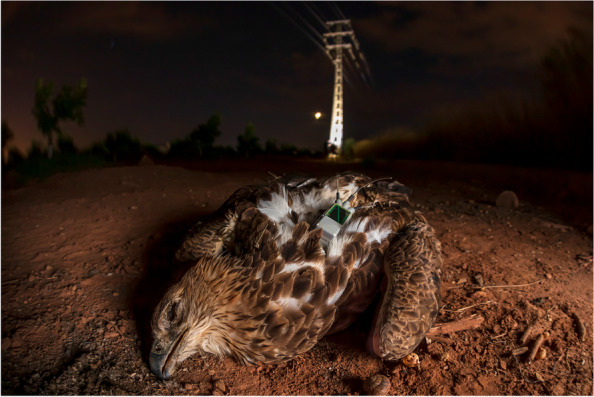
Conservation of the Bonelli's eagle: A GPS transponder allows researchers to identify the power lines a Bonelli's eagle had visited before her death. Attribution: Roberto García-Roa

Roberto García-Roa, an evolutionary biologist and conservation photographer affiliated with the University of Lund in Sweden, submitted the runner-up for 'Protecting our planet'. The photo tells the story of "Bruma", a Bonelli's eagle who sadly died from electrocution caused by a power line. García-Roa says, "The Bonelli's eagle population is declining, and they are now considered endangered in the European Union. GPS transponders track the eagles' movements, helping researchers identify high-mortality areas associated with power lines. In the image, Bruma, who was tagged with a GPS transmitter, lies lifeless after being electrocuted. Information collected by the tracker enabled scientists and authorities to identify the power lines Bruma had visited before her death. This information is crucial for modifying the power lines to prevent other animals from suffering the same fate. Such measures are critical, as thousands of birds die from electrocution in Europe every year." *BMC Ecology and Evolution* Senior Editorial Board Member Josef Settele comments that the photo “shows that interaction of humans and wildlife often is not easy and may have many effects; but there is no way out than looking for solutions—and here the GPS tracks enabled scientists and authorities to identify critical power lines—an information crucial for modifying the power lines to prevent further casualties.” (Fig. 7).

### Life close-up: best in category

Dr Abhijeet Bayani, a biologist from the Indian Institute of Science, captured the winning image for the 'Life close-up' category. Fig trees, crucial in tropical ecosystems as they provide food for many animals throughout the year, have evolved to have flowers inside their fruit. The only way a fig can be pollinated is with the help of minuscule fig wasps, which burrow inside and lay their eggs within. The relationship between figs and fig wasps is highly interdependent—both rely on each other to complete their life cycles, making it a classic example of co-evolution. The female wasps enter the fig, lay eggs, pollinate it and eventually die within the fig. Once the eggs hatch, the male wasps mate with the newly hatched female wasps and create an exit tunnel for the females to leave and locate new figs. Some fig wasps are essential for pollinating the figs, while others, like the non-pollinating wasp captured in Dr Abhijeet Bayani's winning photo, have evolved a specialised ovipositor to cheat the fig, which is used to bore through the surface of the fruit from the outside and deposit eggs inside. Their offspring then develop inside the safety of the fig, seemingly without benefiting the fig tree itself.

**Fig. 8:**
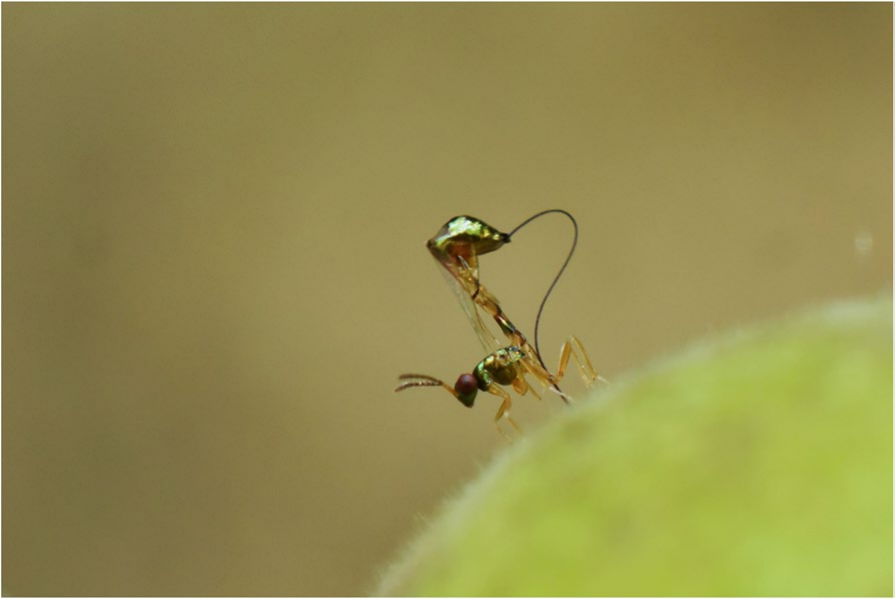
A concerned freeloader: A non-pollinating fig wasp uses its specialised ovipositor to deposit eggs inside a fig. Attribution: Abhijeet Bayani

Dr Bayani remarks, "The non-pollinating fig wasp is one of the most fascinating insects I have observed in my career. These wasps have evolved a complex mechanism to lay eggs inside a fig, but they face unimaginable challenges. On the fig surface, they encounter predatory threats from ants, spiders, and mantids. These predators can catch them while their ovipositor is inserted deep inside the fig, so the wasps must lay eggs quickly and fly away. They also need to be precise to avoid disrupting the proportion of pollinating wasps inside the fig, as the new generation depends on the males of pollinating wasps to exit the fig." *BMC Zoology* Senior Editorial Board Member Brock Fenton adds, "Given how difficult it is to even spot a tiny fig wasp, which measures just a couple of millimetres, taking this photo would have been a real challenge. Capturing this brief moment tells a great story." (Fig. 8).

### Life close-up: runner-up

**Fig. 9:**
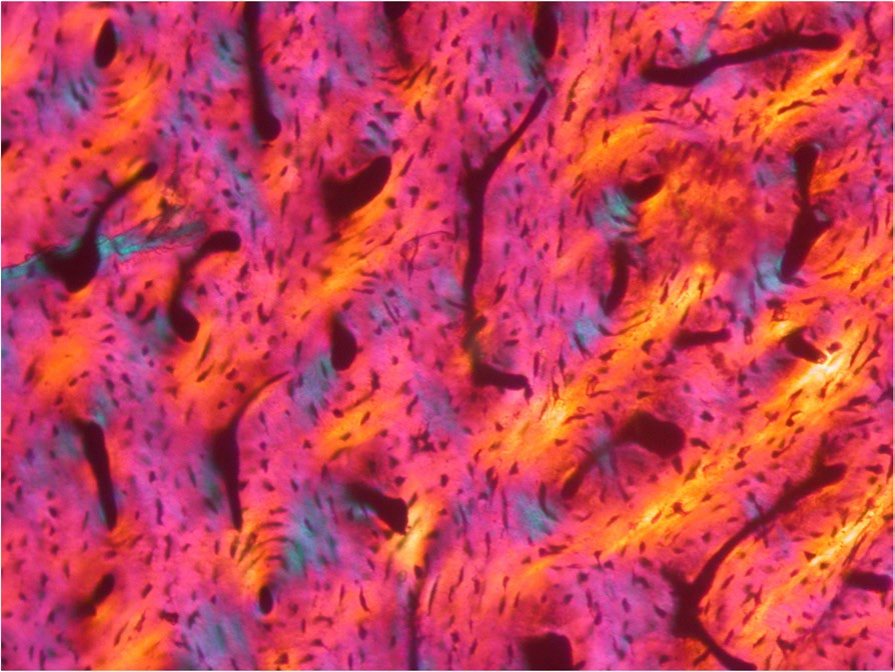
Inside a dinosaur bone: Unlocking the life history of Megapnosaurus. Attribution: Anusuya Chinsamy-Turan

Professor Anusuya Chinsamy-Turan, a palaeontologist from the University of Cape Town, submitted the runner-up for 'Life close-up'. Using a microscope, Chinsamy-Turan demonstrates how fossilised bones can provide valuable information about long-extinct species. "Fortunately, even after millions of years, the microscopic structure of fossilised bone remains intact," comments Chinsamy-Turan. "When viewed under a petrographic microscope, this image of a thin section of a femur of Megapnosaurus, a predatory dinosaur from Southern Africa, reveals important information about the biology of this ~ 190-million-year-old animal. The large black areas once contained blood vessels, nerves, and other connective tissue, while the smaller black specks are the spaces in which bone cells were once located. After death, organic tissue decomposes, and the empty spaces are infilled with sediment and minerals. The vibrant colours visible in the image provide insight into the organisation of the apatite, which is the mineral component of bone that survives fossilisation. In life, collagen and apatite are intimately associated, and although collagen is no longer present, the overall arrangement of the apatite suggests that the bone was deposited rapidly." Studying the microstructure of fossilised bone can provide valuable insight into the life history of dinosaurs and other extinct vertebrates. This can include details about the sex, growth rate, presence of disease, and the potential impact of environmental factors on the growth of the extinct animal (Fig. 9).

## Conclusions

We thank everyone who participated in this year's joint *BMC Ecology and Evolution* and *BMC Zoology* image competition. The competition produced a spectacular collection of images showcasing nature's wonders and the remarkable work conducted by ecologists, evolutionary biologists, zoologists and palaeontologists around the globe.

All figures in this Editorial are released under a Creative Commons Attribution Licence (CC BY) to ensure credit with proper attribution [11]. If you wish to re-distribute or re-use any Figures published in this editorial, please credit individual winners as the image licensee.
